# Surface expression of hippocampal NMDA GluN2B receptors regulated by fear conditioning determines its contribution to memory consolidation in adult rats

**DOI:** 10.1038/srep30743

**Published:** 2016-08-04

**Authors:** Yan-Yan Sun, Wei Cai, Jie Yu, Shu-Su Liu, Min Zhuo, Bao-Ming Li, Xue-Han Zhang

**Affiliations:** 1 Institute of Neurobiology and State Key Laboratory of Medical Neurobiology, Institutes of Brain Science, Fudan University, Shanghai 200032, China; 2 Center for Neuron and Disease, Frontier Institutes of Science and Technology, Xi’an Jiaotong University, Xi’an 710049, China; 3 Department of Physiology, Faculty of Medicine, University of Toronto, 1 King’s College Circle Toronto, Ontario M5S 1A8, Canada; 4 Center for Neuropsychiatric Diseases, Institute of Life Science, Nanchang University, Nanchang 330031, China

## Abstract

The number and subtype composition of N-methyl-d-aspartate receptor (NMDAR) at synapses determines their functional properties and role in learning and memory. Genetically increased or decreased amount of GluN2B affects hippocampus-dependent memory in the adult brain. But in some experimental conditions (e.g., memory elicited by a single conditioning trial (1 CS-US)), GluN2B is not a necessary factor, which indicates that the precise role of GluN2B in memory formation requires further exploration. Here, we examined the role of GluN2B in the consolidation of fear memory using two training paradigms. We found that GluN2B was only required for the consolidation of memory elicited by five conditioning trials (5 CS-US), not by 1 CS-US. Strikingly, the expression of membrane GluN2B in CA1was training-strength-dependently increased after conditioning, and that the amount of membrane GluN2B determined its involvement in memory consolidation. Additionally, we demonstrated the increases in the activities of cAMP, ERK, and CREB in the CA1 after conditioning, as well as the enhanced intrinsic excitability and synaptic efficacy in CA1 neurons. Up-regulation of membrane GluN2B contributed to these enhancements. These studies uncover a novel mechanism for the involvement of GluN2B in memory consolidation by its accumulation at the cell surface in response to behavioral training.

The N-methyl-D-aspartate receptor (NMDAR) is widely known to be the synaptic coincidence detector that is not only required for certain types of learning^1^,^2^ but also essential for controlling synaptic plasticity^3^ and gating memory formation^4^,^5^,^6^,^7^,^8^. Functional NMDARs consist primarily of two GluN1 subtypes and two GluN2 subtypes, with GluN2A and GluN2B subtypes being the most common NMDAR subtypes found in cortical and hippocampal regions of the adult brain. It is generally accepted that increased incorporation of GluN2B into NMDAR complexes enables a longer time window for detecting synaptic coincidence, which benefits memory formation. However, despite gain-of-function and loss-of-function genetic evidence linking the GluN2B subtype to hippocampus-dependent learning and memory^8^,^9^, GluN2B is not necessary for hippocampus-dependent memory in some case (e.g., memory elicited by a single conditioned stimulus-unconditioned stimulus (CS-US) pairing)^10^,^11^. Therefore, the precise role of GluN2B in memory formation, such as in the phases of acquisition and consolidation, is not fully understood.

The hippocampal CA1 region is crucial for converting new memories into long-term memories, a process known as “consolidation”^12^. Study using CA1-specific GluN1 knockout mice demonstrates that NMDAR-mediated synaptic transmission is crucial for memory consolidation^7^. Several lines of evidence demonstrate that GluN2B is implicated in the consolidation of hippocampus-dependent memory, as demonstrated by the impairment of spatial memory consolidation via the disruption of CaMKII binding to GluN2B^13^ and the GluN2B-mediated synaptic plasticity required for spatial memory consolidation^14^. In addition, an increased amount of synaptic GluN2B promotes synaptic plasticity required by memory consolidation^2^,^14^,^15^,^16^,^17^,^18^. However, the contribution of NMDAR GluN2B receptor to the consolidation of memory is a lack of study.

The number and subtype composition of NMDARs has long been thought to be rather static within mature synapses. However, accumulating evidence indicates that the number of GluN2A and GluN2B subtypes changes following synaptic plasticity^17^ and sensory experience^19^,^20^,^21^. In addition to these well-described changes within mature synapses, emerging evidence suggests that GluN2A or GluN2B expression in the adult cortex and hippocampus can also be dynamically modulated by individual experiences (i.e., enriched environment or social interactions)^22^,^23^.

In the present study, we found that the expression of membrane GluN2B within CA1 was increased after fear conditioning in a conditioning-strength-dependent manner. Furthermore, increase in membrane GluN2B expression contributes to the enhanced activities of cAMP, ERK, and CREB in the CA1 region, as well as enhanced intrinsic excitability and EPSP-Spike (E-S) coupling in CA1 pyramidal neurons. Notably, requirement of GluN2B for memory consolidation depended on the amount of GluN2B on the cell surface. These results provide a new insight regarding the role of the GluN2B subtype in memory formation.

## Results

### Role of GluN2B in the acquisition and consolidation of memory

We previously demonstrated that fear memory is sensitive to the injection of GluN2B antagonists into area CA1 before training with five conditioning trials (5 CS-US), but not with a single conditioning trial (1 CS-US)^10^,^11^. Five conditioning trials elicited more freezing behavior than a single conditioning trial in naïve rats when assessed hippocampus-dependent contextual fear memory at 48 h after training (5 CS-US: 84.7 ± 4.7% freezing, n = 6; 1 CS-US: 72.1 ± 2.9%, n = 9; *p* < 0.05).

**Figure 1:**
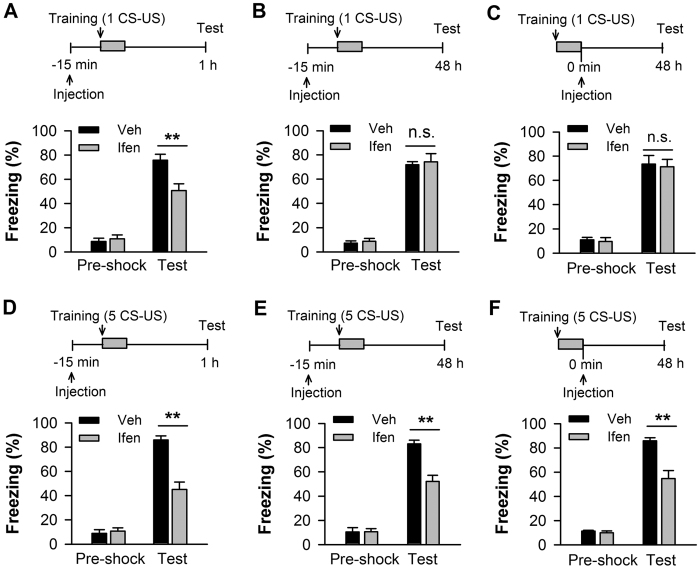
GluN2B is required for the acquisition of contextual fear memory in the CA1 region, but its requirement for memory consolidation depends on conditioning strength. Hippocampal injections of the GluN2B-selective antagonist ifenprodil (ifen) before fear conditioning impaired 1-h short-term memory elicited by a single conditioning trial (1 CS-US). (**B**,**C**) Hippocampal injections of ifenprodil before (**B**) or immediately after (**C**) fear conditioning had no effect on 48-h long-term memory elicited by 1 CS-US. **(D**) Hippocampal injections of ifenprodil before fear conditioning impaired 1-h short-term memory elicited by five conditioning trials (5 CS-US). (**E**,**F**) Hippocampal injections of ifenprodil before (**E**) or immediately after (**F**) fear conditioning impaired 48-h long-term memory elicited by 5 CS-US. *Top panels* indicate the design of the experiments in A–F. **p < 0.01.

To distinguish the role of GluN2B in the acquisition and consolidation of contextual memory, we administered the GluN2B selective antagonist ifenprodil into CA1 prior to training and tested short-term memory at 1 h or long-term memory at 48 h post-conditioning. In addition, we inhibited GluN2B immediately after conditioning and tested long-term memory at 48 h post-conditioning (Fig. 1A–F, *insets*). Conditioning with a single trial (1 CS-US), the rats that received ifenprodil 15 min before training showed significantly reduced freezing behavior when tested at 1 h post-training (p < 0.01; n = 7–8; Fig. 1A), but showed intact freezing behavior at 48 h post-training (p > 0.05; n = 5–7; Fig. 1B) which replicated our previous results^11^,^24^. The rats that received ifenprodil immediately after training also showed intact freezing behavior at 48 h post-training (p > 0.05; n = 5–6; Fig. 1C). In contrast, conditioning with five trials (5 CS-US), the rats that pre-training received ifenprodil showed a significant decrease in freezing behavior when tested at 1 h (p < 0.01; n = 5–7; Fig. 1D) and 48 h post-training (p < 0.01; n = 5–7; Fig. 1E). Post-training infused ifenprodil into CA1 produced a similar detrimental effect on memory at 48 h post-training (p < 0.01; n = 6–8; Fig. 1F). Thus, GluN2B within CA1 is required for the acquisition of contextual fear memory, but it is only necessary for the consolidation of fear memory elicited by five, not by a single conditioning trial.

### Fear conditioning increases amount of membrane GluN2B in CA1

**Figure 2:**
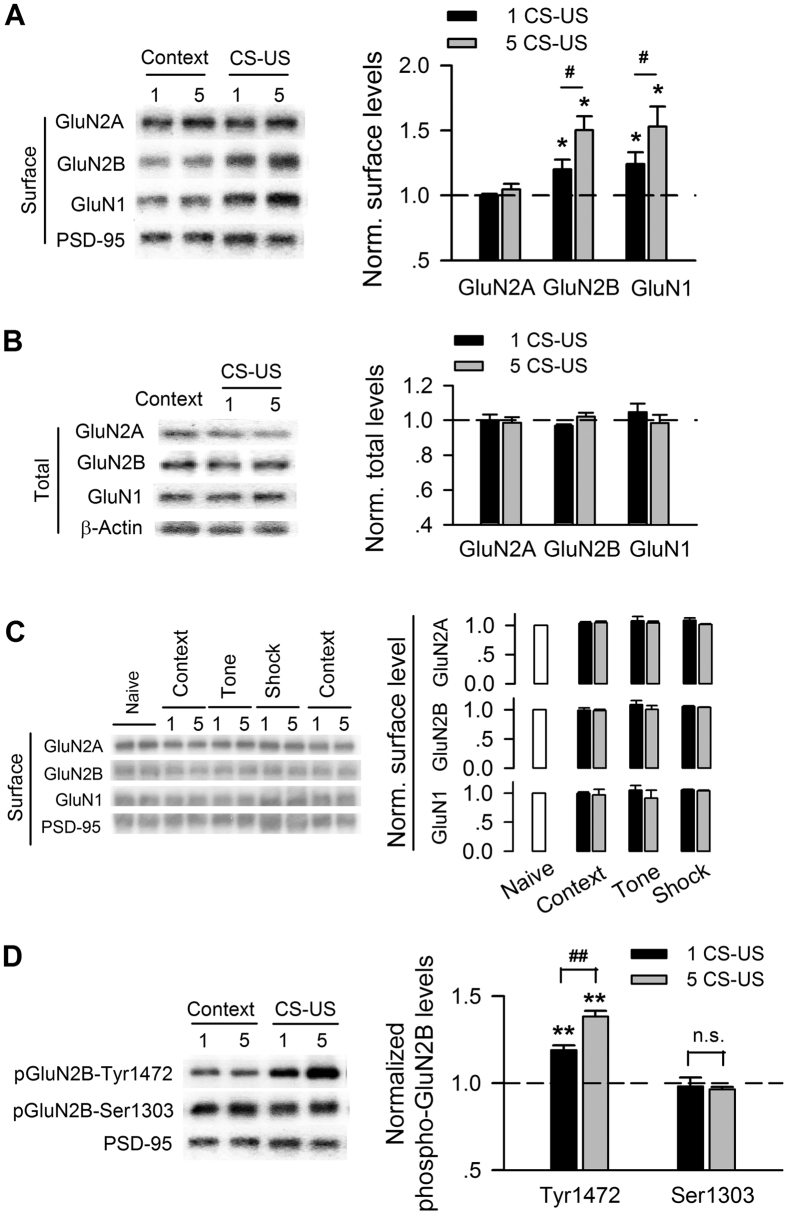
The amount and phosphorylation of membrane GluN2B-containing NMDARs exhibit a conditioning-strength-dependent increase after fear conditioning. (**A**) Immunoblots (*left*) and quantification analysis (*right*) of surface NMDAR subtypes in membrane lysates of CA1 taken from rats unconditioned (context) versus rats conditioned with single (1 CS-US) or five CS-US (5 CS-US), normalized to context control. (**B**) Immunoblots (*left*) and quantification analysis (*right*) of NMDAR subtypes in total lysates of CA1 taken from rats with the same treatments as in (**A**). (**C**) Immunoblots (*left*) and quantification analysis (*right*) of surface NMDAR subtypes in membrane lysates of CA1 taken from naïve, context alone, tone alone, and shock alone rats, normalized to naïve rats. (**D**) Immunoblots (*left*) and quantification analysis (*right*) of GluN2B phosphorylation at Tyr1472 and Ser1303 in membrane lysates of CA1 taken from rats with the same treatments as in (**A**). *p < 0.05, **p < 0.01 *vs*. context-control; ^#^*p* < 0.05, ^##^*p* < 0.01.

We examined the surface expression of NMDAR subtypes after fear conditioning. The rats conditioned with a single CS-US showed a significant increase in the surface expression of the GluN1 (p < 0.05) and GluN2B (p < 0.05) NMDAR subunits in CA1 (n = 3–5 rats for each group), whereas a comparable level of surface GluN2A was observed (p > 0.05; n = 3 for each group; Fig. 2A). Similar changes in the surface expression of GluN1 (p < 0.01), GluN2B (p < 0.01) and GluN2A (p > 0.05) were observed in the rats conditioned with five CS-US (n = 3–5 rats for each group, Fig. 2A). More importantly, rats conditioned with five CS-US showed much larger increases in the amounts of membrane GluN1 (p < 0.05) and GluN2B (p < 0.05) than those conditioned with a single CS-US (Fig. 2A). However, neither single nor five conditioning trials changed the total amount of the GluN1, GluN2A, and GluN2B of NMDAR subtypes (p > 0.05; n = 3 for each group; Fig. 2B). Shock alone, tone alone, or context alone had no impact on the amount of membrane NMDAR subtypes (p > 0.05; n = 3–6 for each group; Fig. 2C).

The surface expression of GluN2B is subject to direct regulation via the phosphorylation of GluN2B by tyrosine kinases^25^ and CaMKII^26^ at specific phosphorylation sites. We assessed the phosphorylation status of membrane GluN2B at Tyr1472 and Ser1303 after fear conditioning. Phosphorylation of GluN2B at Tyr1472 significantly increased after conditioning with both a single (p < 0.01; n = 3 for each group) and five CS-US (p < 0.01; n = 3 for each group; Fig. 2D). Consistent with the surface expression of GluN2B, conditioning with five CS-US induced a larger increase in phosphorylation at Tyr1472 compared with a single CS-US (p < 0.05). However, neither single CS-US nor five CS-US conditioning altered the phosphorylation of GluN2B at Ser1303 (p > 0.05; n = 3 for each group; Fig. 2D). When the time point of the examination was extended to 30 min post-training, increases in the amount and phosphorylation at Tyr1472 of membrane GluN2B were not observed (Supplementary Fig. 1).

Together, these data suggest that fear conditioning induced a rapid and transient increase in the amount of membrane GluN2B-containing NMDARs in area CA1.

### Amount of membrane GluN2B determines its role in memory consolidation

Given the high correlation between the amount of membrane GluN2B and the role of GluN2B in memory consolidation, we sought to pharmacologically change the expression of membrane NMDAR.

**Figure 3:**
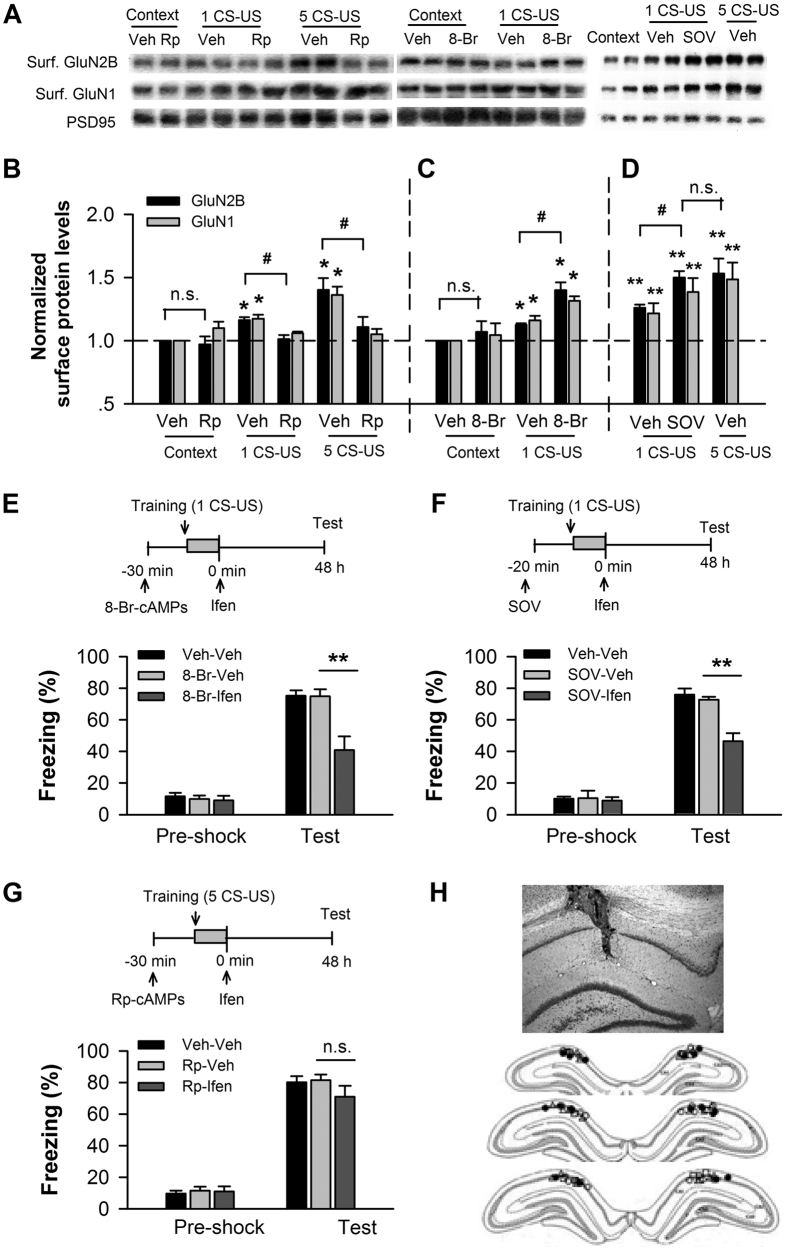
The amount of membrane GluN2B within CA1 determines its contribution to memory consolidation. (**A–D**) PKA signaling is necessary for the increase in the amount of membrane GluN2B-containing NMDARs after fear conditioning. Immunoblots (**A**) and quantification analysis (**B**–**D**) of the GluN2B and GluN1 subtypes of NMDAR in membrane lysates of CA1 taken from conditioned rats with intra-CA1 infusions of vehicle (veh) versus the PKA inhibitor Rp-cAMPs (Rp) (**B**) before training, and taken from conditioned rats with intra-CA1 infusion of veh versus the PKA activator 8-Br-cAMPs (8-Br) (**C**) or sodium orthovanadate (SOV) (**D**), a protein tyrosine phosphatase inhibitor, before training with a single CS-US (1 CS-US), normalized to context-vehicle or context-control. **p < 0.01, *p < 0.05 *vs*. controls; ^#^*p* < 0.05. (E,F) Up-regulating membrane GluN2B expression within CA1, GluN2B is necessary for the consolidation of memory elicited by 1 CS-US. Pre-conditioning infusions of 8-Br-cAMPs (8-Br-ifen) (**E**) or SOV (SOV-ifen) (**F**) coupled with post-conditioning infusion of ifenprodil into CA1 impaired the fear memory elicited by 1 CS-US. *Top panels* indicate the design of the experiments in E and F. **p < 0.01. (**G**) Inhibiting the up-regulation of membrane GluN2B within CA1, GluN2B is not necessary for the consolidation of memory elicited by 5 CS-US. Pre-conditioning infusion of Rp-cAMPs coupled with post-conditioning infusion of ifenprodil (Rp-ifen) into CA1 had no impact on the fear memory elicited by 5 CS-US. *Top panel* shows the design of the experiment. (**H**) A representative coronal section of an infusion site of the co-infusion of SOV with ifenprodil (SOV-Ifen) into CA1 (*upper*). Reconstruction of infusion sites in the CA1 region (*bottom*). Open circles: veh-veh; open squares: SOV-Ifen; open triangles: SOV; filled circles: SOV+Ifen.

The phosphorylation of NMDAR at Tyr1472 has been proposed to be regulated by PKA. To evaluate the possible role of PKA in the up-regulation of membrane GluN2B expression, we micro-infused the PKA inhibitor Rp-cAMPs or activator 8-Br-cAMPs into CA1 before training and examined the expression of membrane GluN2B and GluN1 NMDAR subtypes after training. Western blot analysis showed that inhibition of PKA blocked the up-regulation of membrane GluN2B and GluN1 expression induced by conditioning with a single CS-US (*p* < 0.05 for Rp-cAMPs *vs*. vehicle) or five CS-US (*p* < 0.05 for Rp-cAMPs *vs*. vehicle; Fig. 3B) (n = 3–4 for each group). Conversely, activation of PKA significantly increased the amounts of these subtypes on the membrane after conditioning with a single CS-US (*p* < 0.05 for 8-Br-cAMPs *vs*. vehicle; n = 3–4 for each group), which compared with vehicle control after conditioning with five CS-US (*p* > 0.05; Fig. 3C). Neither Rp-cAMPs nor 8-Br-cAMPs affected the expression of membrane GluN2B and GluN1 subtype in the context-control rats (*p* > 0.05; n = 3–4 for each group; Fig. 3B,C). Furthermore, we micro-infused sodium orthovanadate (SOV) and examined NMDAR subtype expression (Fig. 3D). SOV, a protein tyrosine phosphatase (PTP) inhibitor, increases the level of surface NMDAR by inhibiting tyrosine residue dephosphorylation^27^. As shown in Fig. 3D, pre-training infusion of SOV into CA1 produced a significant increase in the amount of membrane GluN2B and GluN1 subtypes after conditioning with a single CS-US (*p* < 0.05 for SOV *vs*. vehicle; n = 3; Fig. 3D), which is comparable to the increases observed in the vehicle control rats conditioned with five CS-US (*p* > 0.05; n = 3; Fig. 3D).

We then examined whether the memory elicited by a single conditioning trial (1 CS-US) is sensitive to GluN2B inhibition when amount of membrane GluN2B is increased and conversely, whether the sensitivity of five-conditioning-trial-induced memory to GluN2B inhibition is abolished when up-regulation of membrane GluN2B expression is prevented. We increased the expression of membrane GluN2B using 8-Br-cAMPs and SOV and examined effects of ifenprodil on 48-h fear memory elicited by a single CS-US. Behavioral testing showed that co-administration of 8-Br-cAMPs (before training) with ifenprodil (immediately after training) dramatically impaired the 48-h fear memory elicited by a single CS-US (p < 0.01 for 8-Br-ifen *vs*. 8-Br-veh, p > 0.05 for 8-Br-veh vs. veh-veh; n = 5–7 for each group; Fig. 3E). Similarly, co-infusion of SOV (before training) with ifenprodil (immediately post-training) produced a severe deficit in the 48-h fear memory elicited by single CS-US (p < 0.01 for SOV-ifen vs. SOV-veh, p > 0.05 for SOV-veh vs. veh-veh; n = 7–11 for each group; Fig. 3F), which mimicked the effect of ifenprodil on five-CS-US-induced memory. In contrast, co-infusion of Rp-cAMPs (before training) and ifenprodil (immediately post-training) into CA1 had no impact on 48-h fear memory elicited by five CS-US (p > 0.05; n = 5–7 for each group; Fig. 3G). Figure 3H showed a coronal section of an infusion site of the infusion of SOV-Ifen into CA1 (*upper*) and a reconstruction of infusion sites in the CA1 region (*bottom*). These data indicate that the increased amount of membrane GluN2B by fear conditioning requires PKA signaling and that the requirement of GluN2B for memory consolidation depends on its amount on the cell surface.

### Enhanced cAMP, ERK, and CREB activity after fear conditioning

**Figure 4:**
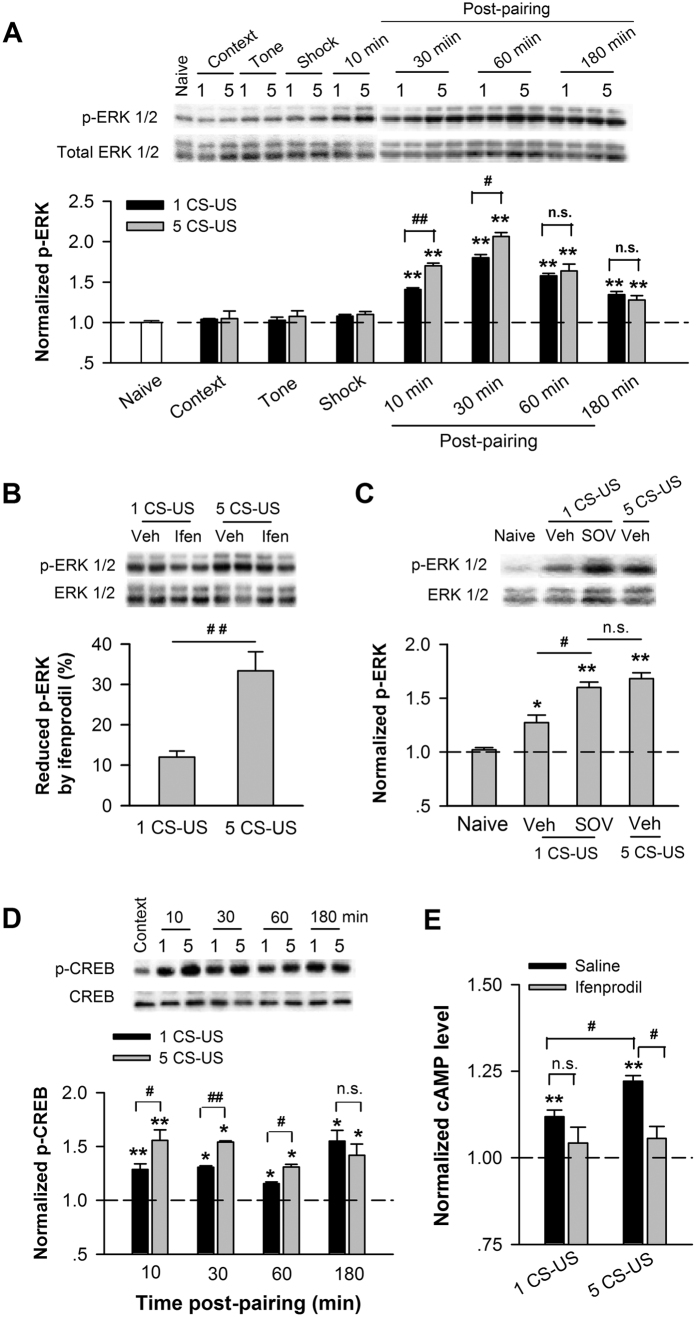
Increased amount of membrane GluN2B enhances the activity of the cAMP, ERK, and CREB signaling pathway in CA1 after fear conditioning. Time course of ERK activation after fear conditioning. Immunoblots (*upper*) and quantification analysis (*bottom*) of ERK phosphorylation (p-ERK) in CA1 lysates taken from naïve, context alone, tone alone, shock alone, and conditioned rats, normalized to naïve rats. (**B,C**) Increased amount of membrane GluN2B enhances ERK activation. (**B**) Micro-injection of ifenprodil before training reduced ERK phosphorylation in area CA1. (**C**) Micro-injection of SOV before training with 1 CS-US increased ERK phosphorylation in CA1, normalized to vehicle-control or naïve rats. *Top panels* indicate the immunoblots. (**D**) Time course of CREB activation after fear conditioning. Immunoblots (*top*) and quantification analysis (*bottom*) of CREB phosphorylation (p-CREB) in CA1 lysates taken from unconditioned and conditioned rats, normalized to unconditioned context-control rats. (**E**) Effect of ifenprodil on the cAMP level in the CA1 region after fear conditioning. Quantification analysis of cAMP concentration in CA1 lysates extracted from unconditioned rats with infusion of saline (context) and conditioned rats with intra-CA1 infusion of saline versus ifenprodil before training, normalized to context-control rats. *p < 0.05 and **p < 0.01 *vs*. control, ^#^*p* < 0.05, ^##^*p* < 0.01.

The Ca^2+^, cAMP, ERK/MAP kinase (MAPK) signal pathways are critical for hippocampus-dependent memory consolidation, a process that depends on cAMP response element-binding protein (CREB) mediated transcription^28^. The ERK pathway is an essential component of NMDAR signal transduction controlling synaptic plasticity and behavioral memory. We first examined the phosphorylation of ERK (p-ERK) in the CA1 at different time points after training using western blot. Conditioned rats exhibited an increased level of p-ERK in CA1 compared with control rats, such as naive, context, tone, and shock (n = 3–4 for each group) examined at 5–10 min post-training (p < 0.01; Fig. 4A). One-way ANOVA showed main effects of p-ERK level at different time points after a single CS-US (p < 0.01) or five CS-US (p < 0.01; Fig. 4A). *Pos hoc* analysis revealed that the p-ERK reached a peak level at 30 min after training (p < 0.01) and returned back at 180 min post-training (p > 0.05 for 180 min vs. 10 min post-training; Fig. 4A). Notably, five CS-US produced a larger increase in p-ERK level than single CS-US till to 30 min post-training (p < 0.01). No significant difference in p-ERK level was observed between single and five CS-US conditioning at 60 min (p > 0.05) and 180 min (p > 0.05) post-training (Fig. 4A), respectively. To access whether the increased amount of membrane GluN2B affects the activity of ERK-CREB pathway, we then inhibited GluN2B by micro-injection of ifenprodil into CA1 before training and examined the level of p-ERK after training. Figure 4B showed that ifenprodil blocked much larger percentage of p-ERK in CA1 taken from rats conditioned with five CS-US than with single CS-US (p < 0.01, n = 6–7 for each group; Fig. 4B). Conversely, after up-regulating the expression of membrane GluN2B by injecting SOV before training, single CS-US induced a significant increase in p-ERK level (p < 0.01), where the increased level of p-ERK induced by single CS-US was comparable with that elevated by five CS-US (p > 0.05; n = 3; Fig. 4C). In addition, fear conditioning induced an increase in the level of CREB phosphorylation (p-CREB) at 10 min, 30 min, 60 min, and 180 min after training compared with naïve (p < 0.05; n = 3 for each group; Fig. 4D). Similarly, conditioning with five CS-US induced a more increase in p-CREB than a single CS-US (p < 0.05), and that the significant difference between single and five CS-US extended to 60 min post-training and vanished at 180 min post-training (p > 0.05; Fig. 4D).

Finally, we examined the level of cAMP after fear conditioning using immunoassay kit. As showed in Fig. 4E, the concentration of cAMP in the CA1 was increased after conditioning (p < 0.01), while conditioning with five CS-US induced a more increase in cAMP concentration than a single CS-US (p < 0.05). Furthermore, we inhibited GluN2B by micro-injection of ifenprodil into CA1 before conditioning and examined the level of cAMP after training. Figure 4E showed that ifenprodil blocked a large of percentage of cAMP level in CA1 taken from rats conditioned with five CS-US (p < 0.05 for ifenprodil *vs*. saline). However, there was no significant reduction in cAMP concentration in rats conditioned with a single CS-US (p > 0.05, Fig. 4E) (n = 3 for each group).

Thus, the increased amount of membrane GluN2B after conditioning contributes to the enhancement of cAMP, ERK, and CREB signaling in area CA1, consistent to a recent study on genetic mice, which confirm that GluN2B overexpression in forebrain enhances the phosphorylation levels of ERK and CREB^29^.

### Enhanced NMDAR functions in CA1 neurons after fear conditioning

**Figure 5:**
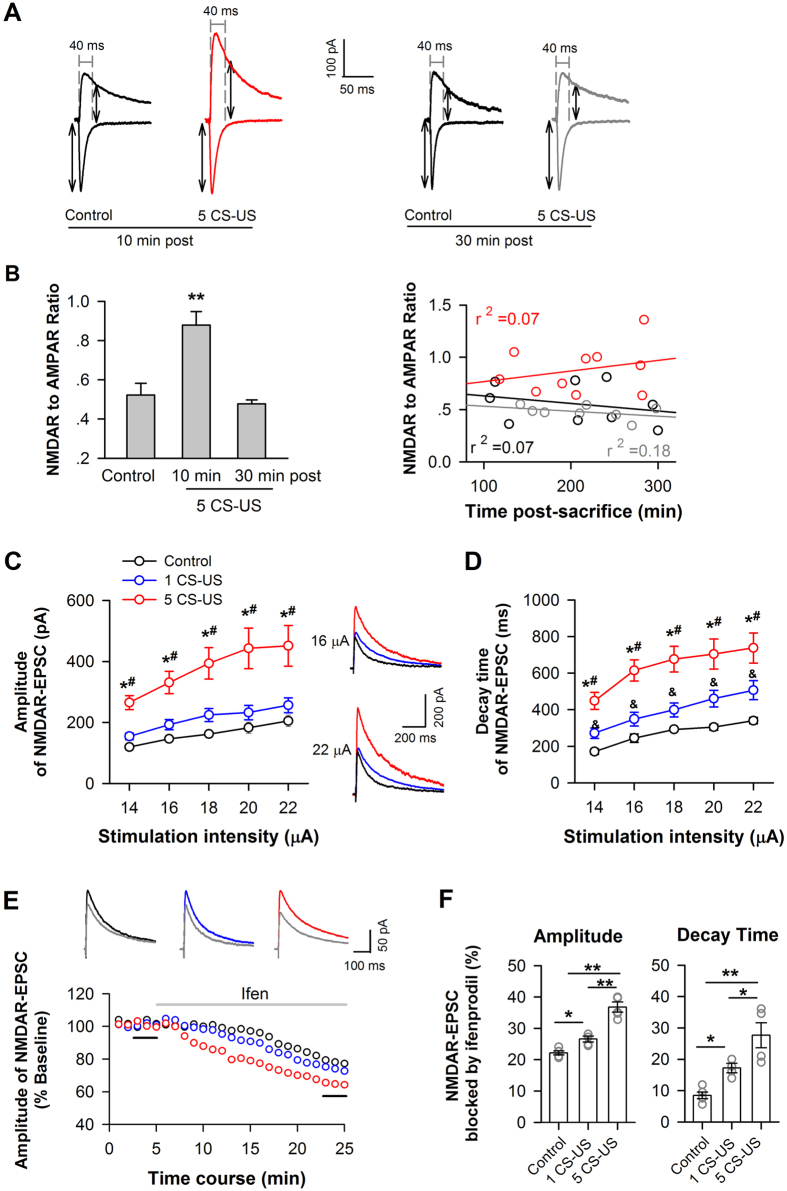
Increased amount of membrane GluN2B enhances NMDAR functions in CA1 neurons after fear conditioning. (**A,B**) Change in the NMDAR/AMPAR current ratio after fear conditioning. CA1 slice was prepared in 10 min or 30 min after conditioning. To measure NMDAR/AMPAR current ratio in CA1 pyramidal neurons, the AMPAR response was quantified as the peak current at a holding potential of −70 mV; the NMDAR response was quantified using a holding potential of +40 mV and was measured 40 ms after stimulation onset. (**A**) Representative evoked EPSCs from context control neurons and 5 CS-US conditioned neurons at these holding potentials. (**B**) Group data for the NMDAR/AMPAR current ratio (*left*). Plots of NMDAR/AMPAR ratio against recording time for individual neurons from slices prepared at 10 min (*red circles*) or 30 min (*grey circles*) post-conditioning with 5 CS-US or context control (*black circles*) (*right*). **p < 0.01 *vs*. context or 30 min post, (**C,D**) Enhanced NMDAR functions after fear conditioning. (**C**) Summarized input-output curves of NMDAR-EPSCs in response to a series of stimulation intensities in CA1 pyramidal neurons from context-control (control) versus conditioned rats. *Insets,* NMDAR-EPSC traces represent the average of seven consecutive recordings from a neuron in CA1 slices taken from a context-control rat (black trace) versus a rat conditioned with single (blue trace) or five CS-US (red trace). (**D**) Summarized decay time of NMDAR-EPSCs, using the same data set as in B. *p < 0.05 *vs*. control; ^#^*p *< 0.05 *vs*. 1 CS-US; ^&^*p *< 0.05 *vs*. control. (**E,F**) Blockade of GluN2B partially inhibited NMDAR-mediated EPSCs. (**E**) The time course of changes in NMDAR-EPSC amplitude before and during the application of ifenprodil (indicated by grey line) in a context control neuron (black) versus a neuron conditioned with single (blue) or 5 CS-US (red). *Insets*: each trace represents the average of seven consecutive recordings before (black trace) and during ifenprodil (grey trace) at the time points indicated by the short black lines. (**F**) Summary data for the effect of ifenprodil on the amplitude (*left*) and decay time (*right*) of NMDAR-EPSCs. *p < 0.05, **p < 0.01.

To examine changes in ionotropic glutamate receptor signaling in CA1 principle neurons after fear conditioning, we quantified the ratio of NMDAR to AMPAR currents in CA1 pyramidal neurons of hippocampal slices taken from conditioned versus context control rats, using conventional whole-cell recording technique. When obtain a stable synaptic response at Vh of −70 mV, which current peak is around 200 pA through adjusting the place of stimulation electrode and the intensity of stimulation at the range of 10–30 μA, Vh was then changed step-by-step to +40 mV. The ratio of peak EPSC at Vh of +40 mV at 40 ms after stimulation (NMDA receptor-mediated current) over the peak current at Vh of −70 mV (AMPAR-mediated current) was calculated (Fig. 5A). *Left panel* in Fig. 5B showed that conditioned neurons (n = 10 cells/6 rats) exhibited a significantly increased NMDAR/AMPAR ratio, compared with context-control neurons (n = 9 cells/6 rats) when hippocampal slices were prepared at 5–10 min post-training, indicative of an increase in overall NMDAR function (p < 0.01; Fig. 5B, *left panel*). However, when slices were prepared at 30 min post-training, no change in NMDAR/AMPAR ratio was observed between conditioned (n = 9 cells/5 rats) and context-control neurons (p > 0.05; Fig. 5B, *left panel*), which is in line with the amount and phosphorylation of membrane GluN2B at 30 min post-training (Supplementary Fig. 1). Moreover, analysis of linear regression demonstrated that NMDAR/AMPA ratio was not affected by time after slice-preparation in conditioned and control neurons (p > 0.05, liner regression; Fig. 5B, *right panel*).

Next, we examined NMDAR-mediated currents in CA1 pyramidal neurons. As shown in Fig. 5C, the amplitude of the NMDAR-EPSCs induced by a series of stimulus intensities were markedly increased in neurons conditioned with five (p < 0.05; n = 6 cells/4 rats) compared with the amplitudes in control neurons (n = 7 cells/6 rats; Fig. 5C). The decay time of the NMDAR-EPSCs increased considerably in the neurons conditioned with five CS-US compared with those conditioned with single CS-US (p < 0.05) or context control neurons (p < 0.01) (Fig. 5D). Although we found no difference in current amplitude of between single- CS-US conditioned neurons (p > 0.05; n = 6 cells/4 rats) and control neurons, the decay time of NMDAR currents from single-CS-US conditioned neurons was 1.4-fold longer than that of control neurons (p < 0.05, Fig. 5D). AMPAR-EPSCs were not changed in five-CS-US-conditioned neurons (p > 0.05, supplementary Fig. 4).

Finally, the effect of blocking GluN2B on NMDAR-EPSCs was examined. Compared with baseline (0–3 min before application of ifenprodil), the amplitude of the NMDAR-EPSCs was significantly reduced after an 18–20 min perfusion of ifenprodil (3 μM) in both conditioned (1 CS-US: 73.9 ± 0.9% of baseline; n = 4 cells/4 rats; 5 CS-US: 63.6 ± 1.4% of baseline; n = 5 cells/4 rats) and control neurons (77.8 ± 0.6% of baseline; n = 5 cells/4 rats; Fig. 5F). One-way ANOVA showed a main effect of ifenprodil on the amplitude (p < 0.01) and decay time (p < 0.01) of NMDAR-EPSCs (Fig. 5F). *Post hoc* analysis confirmed that the larger NMDAR-EPSC amplitudes and decay times were blocked by ifenprodil in the five CS-US conditioned group compared with the other two groups (p < 0.05), which is the same result that was found for the single CS-US conditioned group compared with the control group (p < 0.05; Fig. 5F), suggesting the increased GluN2B incorporating into synaptic sites and functioning.

These results indicated that the up-regulation of membrane GluN2B after fear conditioning enhanced NMDAR-mediated synaptic response in CA1 pyramidal neurons, consistent with the studies on genetic mice^8^,^30^.

### Increased neuronal excitability in CA1 neurons after fear conditioning

**Figure 6:**
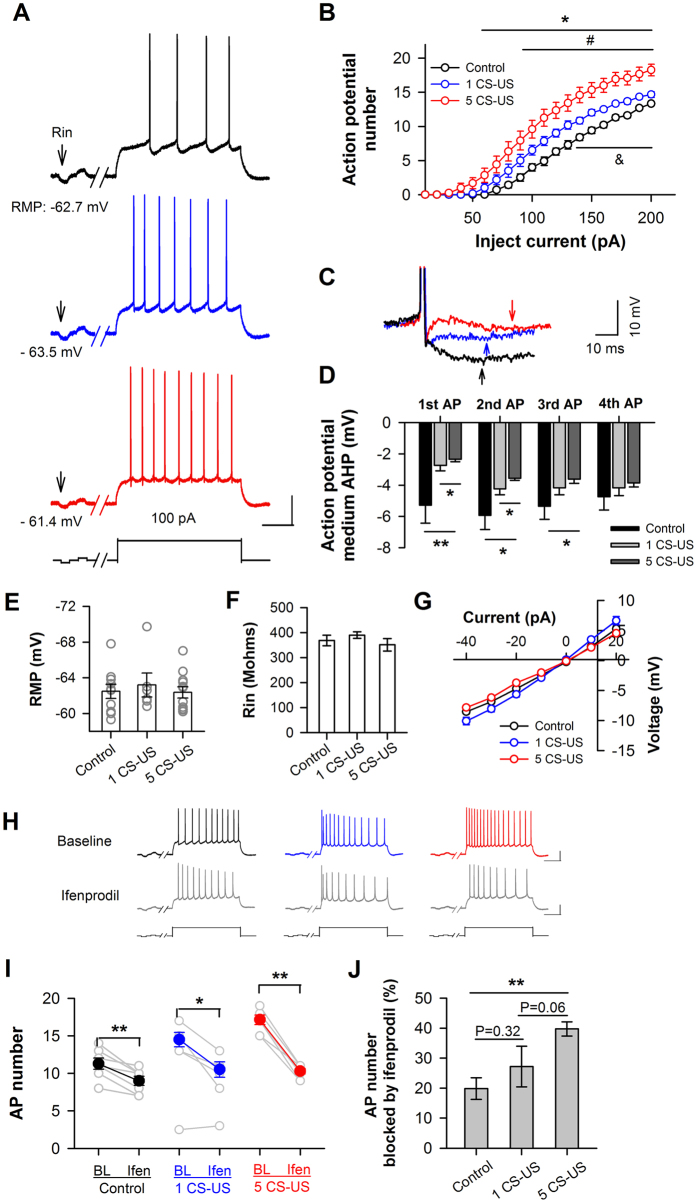
Increased amount of membrane GluN2B enhances intrinsic excitability of CA1 neurons in response to somatic depolarization after fear conditioning. (**A,B**) The number of action potential in CA1 neurons was increased after fear conditioning. (**A**) Representative sweeps of action potentials (APs) elicited by a 100-pA depolarization current in a CA1 pyramidal neuron from rat unconditioned (black) versus conditioned with single (blue) or 5 CS-US (red). RMP, resting membrane potential. Arrows indicate a hyperpolarization current to monitor input resistance (Rin). Calibration: 30 mV, 200 ms. (**B**) Averaged AP number elicited by a sequence of depolarization pulses ranging from 0–200 pA. *p < 0.05 for 5 CS-US *vs.* context control, ^#^*p* < 0.05 for 1 CS-US *vs*. context-control, and ^&^*p* < 0.05 for 1 CS-US vs. 5 CS-US. (**C,D**) (**C**) Detail of the first action potential fired by the examples in (**A**). The arrows are the point where after-hyperpolarization (AHP) measurements were made (20–50 ms after each action potential). The black trace corresponds to the control neuron; the blue trace, to a single CS-US conditioned neuron; and the red trace, to a five CS-US conditioned neuron; (**D**) Summary of the medium AHP (mAHP) voltage for each of the first 4 APs in unconditioned and conditioned neurons, using the same data set as in B. *p < 0.05, **p < 0.01. (**E–G**) Resting membrane potential (RMP) (**E**), input resistance (Rin) (**F**), and current-voltage relationship curves (**G**) were not affected by fear conditioning, using the same data set as in B. (**H–J**) Blockade of GluN2B decreased the number of APs. (**H**) Examples of responses to a 160-pA depolarization current pulses (*bottom*) before (*BL*) and during the application of ifenprodil (*Ife*n) in pyramidal neurons of CA1 slices taken from context-control rats (*black*) versus rats conditioned with single (*blue*) or 5 CS-US (*red*). Calibrations: 30 mV, 200 ms. (**I**) Change in AP number before (*BL*) and during ifenprodil (*Ifen*) application. Data obtained from the same neuron are linked by lines. *p < 0.05, **p < 0.01, paired *t*-test. (**J**) Summary of the effect of ifenprodil on AP number. **p < 0.01.

It has been documented that enhanced CREB increases the excitability of neurons and primes memory consolidation^31^,^32^. To examine whether the excitability of CA1 pyramidal neurons was increased after fear conditioning, we quantified the number of action potential (AP) using current-clamp recording. As shown in Fig. 6G, we did not observe any significant differences in the resting membrane potential (one-way ANOVA; Fig. 6E), in the input resistance (one-way ANOVA; Fig. 6F), or in the current-voltage curve (two-way ANOVA; Fig. 6G) of the CA1 neurons (p > 0.05). However, the number of AP elicited by a sequence of depolarization current injections (-40 pA to 200 pA) was significantly more in the neurons trained with five CS-US (p < 0.05 from 60 pA to 200 pA current injections; n = 12 cells/4 rats) or a single CS-US (p < 0.05 from 90 pA to 200 pA current injections; n = 6 cells/3 rats) than in the control neurons (n = 10 cells/3 rats; Fig. 6B). Increase in the number of APs in the 5 CS-US trained neurons was much greater than in the single CS-US trained neurons (p < 0.05 from 140 pA to 200 pA; Fig. 6B), confirming an increase in the intrinsic excitability of CA1 pyramidal neurons after conditioning. Notably, the increase in the firing frequency in response to a depolarization current was associated with reduced medium after-hyperpolarization (mAHP) (deviation from the threshold during 20–50 ms after each AP) (p < 0.05; Fig. 6D).

To examine whether an increased amount of membrane GluN2B-containing NMDARs contributes to neuronal excitability, we examined the effect of ifenprodil on the AP number. The 15–25 min bath application of ifenprodil (3 μM) significantly suppressed the AP number elicited by a 160-pA depolarization current injection in neurons trained with five CS-US (changed from 17.1 ± 0.6 to 10.4 ± 0.3; p < 0.01, paired t-test; n = 7 cells/4 rats) or a single CS-US (changed from 14.5 ± 0.9 to 10.5 ± 1.0; p < 0.01; n = 4 cells/3 rats) versus untrained rats (changed from 11.6 ± 0.3 to 9.0 ± 0.6; p < 0.01; n = 7 cells/3 rats; Fig. 6I). One-way ANOVA revealed a main effect of ifenprodil on AP number (p < 0.01; Fig. 6J). *Post hoc* analysis confirmed that a larger percentage of the spike number was blocked by ifenprodil in the five CS-US conditioned group compared with the other two groups, while no significant difference was found between the single CS-US and the control groups (p = 0.32).

**Figure 7:**
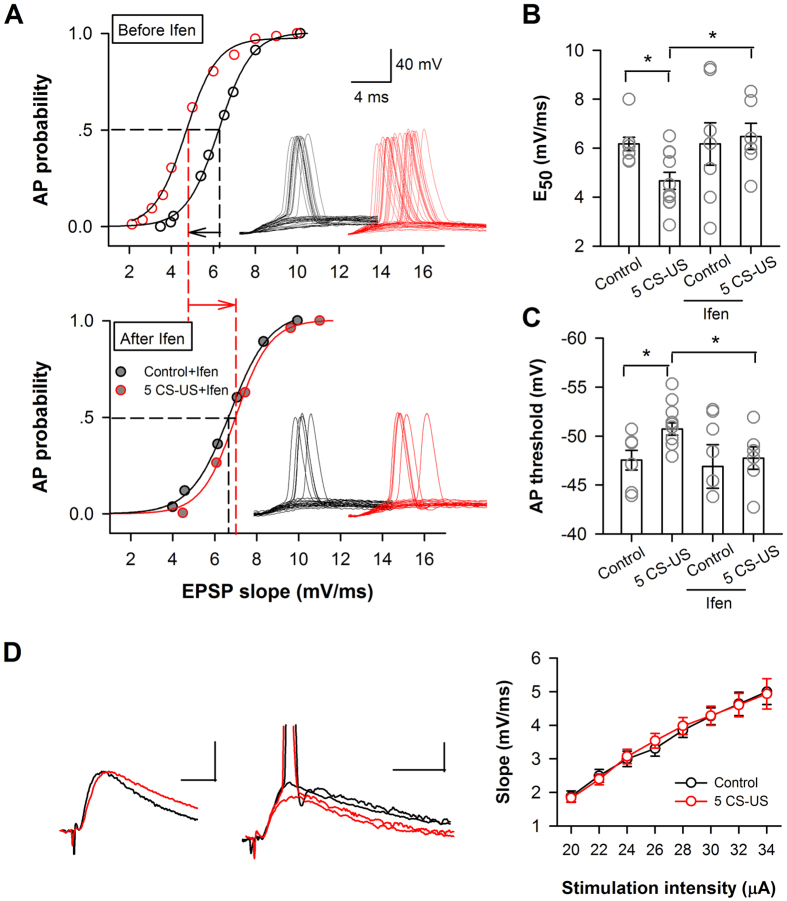
Increased amount of membrane GluN2B enhances E-S coupling of CA1 neurons after fear conditioning. (**A**) E-S coupling studies in context-control rats (control) or conditioned rats (5 CS-US) in the absence (*upper*) and presence (*bottom*) of ifenprodil (*ifen*). Unit E-S curves of a conditioned neuron (*red*) and a context-control neuron (*black*). The curves represent the best fit with a sigmoidal function. The sigmoid fits of the data for a conditioned neuron (*red*) illustrated a leftward shift (black leftward arrow) in firing probability before ifenprodil application (p < 0.05, Kolmogorov-Smirnov test) and a rightward shift (red rightward arrow) after ifenprodil application (p < 0.05, Kolmogorov-Smirnov test). *Inserts*, representative traces of EPSC and action potential following increasing synaptic stimulation, recorded from a control (*black*) and a conditioned (*red*) neuron. (**B**) Individual and summary of the values of E_50_ before and after bath application of ifenprodil (Ifen). E_50_ represents the efficacy of E-S coupling, as defined by the EPSP slope that initiates spiking with a probability of 0.5. (**C**) Individual and summary data of the AP initiation threshold before and after bath application of ifenprodil (Ifen). The threshold was determined by the membrane potential at the peak of the maximal subthreshold EPSP, using the same data set as in (**B**). (**D**) No change in the slope of the linear component of the E-S curve between control neurons and conditioned neurons. *Left*, representative EPSPs and action potential traces from a control neuron and a conditioned neuron. *p < 0.05. Calibrations: 1 mV, 10 ms.

Next, we accessed whether the intrinsic excitability changes in conditioned neurons alter the input-output function of neurons, an essential property for information processing during learning. We measured the relationship between the excitatory postsynaptic potential (EPSP) slope and spike probability following different synaptic stimulation intensities (the E-S curve) in conditioned and context-control neurons. As showed by the example recordings in the upper panel of Fig. 7A, the curve for E-S coupling was left-shifted in a five CS-US conditioned neuron relative to the curve of a context control neuron (p < 0.01, Kolmogorov-Smirnov test). The enhanced E-S coupling is consistent with an increased amount of membrane GluN2B in these neurons because blocking GluN2B by 15–25 min perfusion of ifenprodil (3 μM) led to a rightward shift of the E–S coupling curve in these both groups and eliminated the discrepancy between these two groups of neurons (p > 0.05, Kolmogorov–Smirnov test; Fig. 7A, *bottom panel*). The EPSP slope that yielded a 50% probability of spiking (E_50_) was determined for each neuron recorded (Fig. 7B). The average value of E_50_ was significantly different between the conditioned (n = 10 cells/7 rats) and the control neurons (p < 0.05; n = 6 cells/4 rats). The increased excitability of the CA1 pyramidal neurons was also reflected by the lower AP initiation threshold in the conditioned neurons than in the control neurons (p < 0.05; Fig. 7C). Such changes in threshold were abolished by ifenprodil as well (p > 0.05; 5 CS-US: n = 6 cells/4 rats; control: n = 7 cells/4 rats; Fig. 7B,C). However, the initial slope of EPSP was not changed (p > 0.05, two-way ANOVA; Fig. 7D), indicating that 5 CS-US conditioning did not change basal excitatory synaptic transmission^33^ and inhibition^34^,^35^,^36^. Thus, the E-S potentiation that we observed in 5 CS-US conditioned neurons is probably a result of changes in the intrinsic electrical properties of postsynaptic neurons. Therefore, CA1 neurons from 5 CS-US conditioned rats demonstrate stronger E-S coupling.

Taken together, these data suggest that the increased GluN2B expression enhanced the excitability of CA1 pyramidal neurons in response to both somatic and synaptic stimulation.

## Discussion

The main findings of the present study include these three points: (1) GluN2B number on cell surface is training-strength-dependently elevated *in vivo* after fear conditioning in adult rats; (2) GluN2B is only required for the consolidation of memory elicited by strong training, not by weak training in CA1; (3) contribution of GluN2B to memory consolidation is determined by its amount on the cell surface in adult CA1.

These results suggest that GluN2B within CA1 could be involved in gating memory formation under strong training. Interfering GluN2B in the short time after training could impair long-term memory elicited by strong training. Furthermore, GluN2B devote in gating memory formation by accumulating to cell surfaces in response to behavioral training. The underline mechanism may involve the enhancement of neuronal excitability in CA1 neurons, that is, increased GluN2B expression could enable and/or facilitate training-induced synaptic plasticity, which favoring memory retention.

Genetic or pharmacological evidence has revealed the important role of GluN2B in learning and memory^8^,^9^,^10^,^11^,^24^. In the present study, we inhibited GluN2B by intra-CA1 infusing the GluN2B-selective antagonist ifenprodil prior to behavioral training and examined short-term memory at 1 h and long-term memory at 48 h after training (Fig. 1A,B,D,E). Furthermore, we administered ifenprodil immediately after behavioral training and examined long-term memory to confirm the role of GluN2B in memory consolidation (Fig. 1C,F). Thus, we precisely separated the contribution of GluN2B to the acquisition and consolidation of memory using two training paradigms, and our results indicated that GluN2B-involved short-term memory elicited by weak training could not be converted to long-term memory. In support of it, we found that pre-training inhibiting GluN2B had no effect on CREB phosphorylation (p-CREB) examined at 3 h after weak training, however, significantly reduced p-CREB was observed after strong training (Supplementary Fig. 2). CREB is a critical component of the molecular switch that controls the conversion of short-term memory into long-term memory. Therefore, hippocampal GluN2B is not required for the consolidation of memory elicited by weak training. For weak-training-elicited memory, hippocampal L-type Ca^2+^ channels engage (Supplementary Fig. 3) and GluN2A is required as well^9^,^11^.

Our present study provides strong and direct evidence of a critical link between GluN2B membrane expression and memory storage in adult rats. We found that after pharmacologically increasing the amount of membrane GluN2B via infusing the PKA activator 8-Br-cAMP or the PTP inhibitor SOV into CA1, the memory elicited by weak training was impaired by post-training infusion of ifenprodil, which mimicked the effect of ifenprodil on the memory elicited by strong training (Fig. 3). Consistently, a recent report showed that the increasing the GluN2A to GluN2B subunit ratio by genetically overexpressing GluN2A constrained memory consolidation in the adult brain^37^. Therefore, the decreasing the GluN2A to GluN2B ratio by increasing the amount of membrane GluN2B may facilitate memory consolidation, as indicated by the present results. Thus, the question raises, how does up-regulation of surface GuN2B expression contribute to memory storage?

Memories are encoded in neuronal networks (memory trace). Biophysical modeling study and animal experiments have shown that neurons are recruited to a memory trace based on their relative CREB activity and neuronal excitability^38^,^39^,^40^. In the present study, strong-trained neurons demonstrate the higher CREB activity (Fig. 4) and excitability (Figs 6 and 7) compared with weak-trained neurons. Strong-trained neurons are thus liable to get recruited in a memory trace, as reported previously in hippocampus^41^,^42^,^43^ and amygdala^44^. Furthermore, our data confirmed that the up-regulated GluN2B after training contributes to the increases in CREB activity and neuronal excitability (Figs 4 and 5). Thus, under strong training, GluN2B-enhanced CREB activity and neuronal excitability play a crucial role in determining which particular neurons are selected or allocated to an encoded memory trace. By contrast, although upregulation of GluN2B in weak-trained neurons also enhanced CREB activity and neuronal excitability, these enhancements may not involve in determining the recruitment of a memory trace neuron. This may be due to a competitive mechanism underlying memory formation, which competition takes place between neurons with high versus low intrinsic excitability^40^. We speculated that the enhanced neuronal excitability after weak training may fail to compete with, whereas the enhanced excitability after strong training may be comparable with, neuronal excitability enhanced by other channels, e.g. L-type Ca^2+^ channels. Long-term fear memory was therefore sensitive to GluN2B inhibition under strong but not weak training (Fig. 1). In support of it, inhibition of L-type Ca^2+^ within CA1 produced a detrimental effect on fear memory elicited by both strong and weak training (Supplementary Fig. 3).

Additionally, the E-S coupling determines the neuronal output. Our analyses of E-S coupling indicate that, in addition to enhancing intrinsic excitability, GluN2B up-regulation strengthens input-output functions of conditioned neurons. Thus, the increased neuronal intrinsic excitability and the decreased firing threshold cause the same-sized EPSP to fire more APs, thereby decreasing CA1 neurons’ threshold for synaptic plasticity and enhancing their capacity for plasticity, which promotes the allocation of fear memory to specific cells^45^. Consistent with this idea, both intrinsic excitability and synaptic plasticity were significantly correlated with behavior such that better memory corresponded with a smaller AHP and enhanced LTP in CA1 cells from conditioned rats^46^,^47^.

Intrinsic excitability is regulated by several mechanisms^48^, including learning. Learning transiently increases the intrinsic excitability of some neurons by downregulating K+ currents which mediate the AHP^49^,^50^,^51^. Also, PKA activity has been suggested to be involved in maintaining the AHP reduction after successful learning^52^. Although we do not have direct evidence for the mechanism underlying enhancement in intrinsic excitability by GluN2B up-regulation, we do demonstrate that the increased firing number accompanied with the reduced AHP amplitude and spike threshold in strong-trained neurons (Fig. 6). The GluN2B-activated cAMP and CREB are likely to contribute to these reductions. PKA is a cAMP-dependent protein kinase and has been shown to effectively reduce the AHP in hippocampal pyramidal neurons^53^. Moreover, it has been proposed that activation of PKA induces internalization of AHP channels in hippocampal neurons^54^. In addition to reducing by PKA, AHP is decreased by CREB, as reported that activation of CREB causes a significant AHP reduction in CA1 pyramidal neurons^32^, as well as in neurons of other brain regions^31^,^44^,^55^.

Our results indicated that surface GluN2B expression was rapidly and transiently increased after fear conditioning, as that increases in amount and phosphorylation of membrane GluN2B were observed at 5–10 min after conditioning (Fig. 2) and that these increases vanished at 30 min after conditioning (Supplementary Fig. 1). Moreover, we did not find an increase in NMDAR/AMPAR ratio measured *ex vivo* at 30 min after conditioning (Fig. 5). In addition, the rapid and transient up-regulation of GluN2B triggered sustained activations of intracellular cascades including ERK and CREB after fear conditioning (lasting to 3 h after conditioning) (Fig. 4). Consistently, transiently increasing excitability is sufficient to make the neuron recruited into memory trace and enhances memory formation, as reported by Yiu AP *et al*.^39^.

Here we observed that only GluN2B membrane expression is subject to regulation by behavioral training — GluN2A is not (Fig. 2). This is in line with the report that the synaptic distribution of surface GluN2B — but not GluN2A — rapid changes following long-term potentiation (LTP) induction and this change appears to be instrumental for LTP expression^17^.

Our results indicate that PKA is necessary for the up-regulation of membrane GluN2B expression after fear conditioning (Fig. 3). Changes in synaptic GluN2B expression are regulated via direct phosphorylation of the GluN2B by both CaMKII and tyrosine kinases at specific phosphorylation sites. In the present study, we found that behavioral training enhanced phosphorylation of GluN2B at Tyr1472 but not phosphorylation at Ser1303 (Fig. 2E). Phosphorylation of GluN2B at Tyr1472 disrupts its binding to the endocytic adaptor AP-2, thereby resulting in inhibition of GluN2B endocytosis and an increased amount of GluN2B-containing NMDARs at synapses^56^,^57^. Tyr1472 is a site for Src/Fyn and that Src/Fyn phosphorylation is critical in the regulation of GluN2B-containing NMDAR activity^58^,^59^. Src/Fyn can be phosphorylated by PKA^58^,^60^. Indeed, we found that inhibiting PKA activity within CA1 blocked the increase in membrane GluN2B expression of conditioned rats (Fig. 3).

In summary, our findings provide a new insight for the role of GluN2B in memory storage in adult CA1. The unique mechanism, by which hippocampal GluN2B is training-strength-dependently involved in memory formation, suggests that GluN2B can flexibly participate in memory formation through a rapid change in its membrane expression following individual experience, and thus indicating that GluN2B within the hippocampus could be a potential target to prevent from the formation of “bad” memory, e.g., in posttraumatic stress disorder (PTSD).

## Materials and Methods

### Animals

Adult male Sprague-Dawley rats (180–220 g) were purchased from SLACCAS (Shanghai, China). Animals were housed under a 12-hr light/dark cycle with a room temperature of 23 ± 1 °C. Food and water were available ad libitum. All experiments were carried out in accordance with the guidelines published in the NIH *Guide for the Care and Use of Laboratory Animals* and approved by the Ethical Committee of Animal Experiments at Fudan University Institute of Neurobiology (Shanghai, China). Efforts were made to minimize the number of animals used and their suffering.

### Surgery

Rats were anesthetized with sodium pentobarbital (40 mg/kg, intraperitoneal (i.p.) injection) and implanted with double guide cannulas (23-gauge) aimed at the CA1 region (bregma +3.6 mm, lateral ±2.0 mm, and depth −1.7 mm from the skull surface). Dummy cannulas, cut 0.5 mm longer than the guide cannula, were inserted into the guide cannulas to prevent clogging and to reduce the risk of infection. Rats received a recovery period of at least 5 days before the behavioral procedures. After all of the behavioral procedures, verification of the cannula placements was made from Nissl-stained coronal sections through CA1.

### Drug administrations

Intra-CA1 infusions were performed using 30-gauge infusion cannulas that extended 1.6 mm beyond the end of the guide cannulas, yielding a total distance of 3.3 mm from the skull surface. All infusions were performed at a rate of 0.5 μl/min for a total volume of 1.0 μl per side. The infusion cannulas remained in place for an additional 2 min to allow the drug solution to diffuse away from the tip of the infusion cannula; the dummy cannulas were replaced immediately after each drug infusion.

We infused the following compounds dissolved in saline: the GluN2B-selective antagonist ifenprodil (2 μg/μl), the PKA inhibitor Rp-cAMPs (2.5 μg/μl), the PKA activator 8-Br-cAMPs (2.5 μg/μl), and the protein tyrosine phosphatase (PTP) inhibitor sodium orthovanadate (SOV; 9 μg/μl) from Sigma-Aldrich. A new sealed vial of the drug was used each time, and each solution used was prepared on the same day and stored at −20 °C until use.

### Behavioral procedures

Fear conditioning took place in an isolated conditioning chamber. Two training paradigms were the same as our previous description with a few changes^10^,^24^. Briefly, for single conditioning trial (1 CS-US) protocol, the rats received a single tone CS (2.2 kHz and 96 dB, 20 s), which co-terminated with a foot shock US (1.0 mA, 2 s). For five conditioning trials (5 CS-US) protocol, the rats were presented with five CS-US conditionings (1.0 mA, 0.5 s for each US), with an inter-conditioning interval of 90 s. The following controls were employed: context alone, exposure to the chamber for the same duration as the conditioning protocols; tone alone, exposure to only the tone delivered by the conditioning protocols; shock alone, quick delivery of a single shock (1.0 mA, 2 s) or five shocks (1.0 mA, 0.5 s) to reduce the duration spent in the chamber. Contextual fear memory was tested at 1 h or 48 h after training; in the testing, the rats were placed into the training chamber and were allowed to stay there for 3 min without tone or foot shock. Fear responses were recorded and analyzed using the video-based FreezeFrame Fear Conditioning System (Med Associates, USA).

### Western blot

The rats were decapitated following an overdose of sodium pentobarbital, and the brains were quickly removed at 5–10 min after training not otherwise mentioned. Bilateral CA1 tissues were quickly dissected and frozen in liquid nitrogen and then transferred to storage at −80 °C until lysis. For whole-cell protein isolation, frozen tissues were sonicated in homogenization buffer containing 20 mM Tris-HCl (pH 7.5), 150 mM NaCl, 1% Triton X-100, 1 mM EDTA, 1 mM PMSF, 4% protease inhibitor (Roche, USA) and 1% phosphatase 1 & 2 inhibitor cocktail (Sigma, USA). The homogenates were centrifuged at 12,000 rpm for 25 min at 4 °C. For membrane protein isolation, the Plasma Membrane Protein Extraction Kit (BioVision, USA) was utilized under guidelines of the instructions. Briefly, frozen tissues were sonicated in homogenization buffer (K268-50-1, BioVision). The homogenates were centrifuged at 700 × g for 10 min at 4 °C. The supernatant was then centrifuged at 10,000 × g for 30 min at 4 °C. After purification procedures, the plasma membrane protein was isolated and dissolved in 0.5% Triton X-100 in PBS and boiled. Proteins were quantified using the Micro BCA protein assay (Thermo Scientific) and normalized to 1.0 μg/μl per sample. Electrophoresis was performed on Tris-HCl resolving gels, and the gels were transferred to PVDF membranes (Roche). The membranes were blocked in 5% nonfat dried milk for 2 h at room temperature to block non-specific binding and then incubated overnight at 4 °C with primary antibodies. Antibodies were diluted in 5% nonfat dried milk in TBST. After thoroughly rinsed with TBST, the membranes were incubated for 2 h at 4 °C with secondary antibodies. Signals were finally visualized using enhanced chemiluminescence (Thermo Scientific), and the blots were exposed onto X-Films. The bands corresponding to phosphorylated (p)-ERK1 and p-ERK2 were pooled when quantifying intensity. To analyze the total ERK (or total CREB) content in the samples, blots for p-ERK (or p-CREB) were incubated in a stripping buffer for 30 min at 50 °C. After rinses, the same blots were incubated with 5% nonfat dried milk and probed with an antibody raised against ERK (or CREB) protein under the same conditions. Analysis of the immunoblot data was performed by scanning the films and determining the band intensities using Adobe Photoshop software. For each rat, the data are presented as the mean from at least three separate experiments.

The antibodies used in the present study included anti-GluN2A (1:500), anti-GluN2B (1:800), anti-GluN2B phosphor Tyr1472 (1:200), anti-GluN2B phosphor Ser1303 (1:1000), anti-pCREB (1:1000) and anti-CREB (1:800) from Millipore; and anti-GluN1 (1:1500; BD Pharmingen), anti-PSD95 (1:4000; Abcam), anti-pERK1/2 (1:1000; Cell signaling), anti-ERK1/2 (1:5000; Sigma), anti-β-actin (1: 20000; Sigma) and peroxidase-conjugated donkey anti-mouse or donkey anti-rabbit antibodies (1:8000; Jackson).

### cAMP detection

The cAMP levels of CA1 tissue were detected using a kit (Direct Immunoassay Kit, BioVision, USA).

### Electrophysiology

Age-matched male Sprague-Dawley rats (180–220 g) were anesthetized with sodium pentobarbital (40 mg/kg, i.p.) at 5–10 min after behavioral training not otherwise mentioned. Transverse hippocampal slices (300 μm in thickness) were rapidly prepared, and whole-cell patch clamp recordings were made from CA1 pyramidal neurons using the previously described method^10^. Briefly, slices removed CA3 region was removed were incubated at 30 °C for at least 1 h and were then transferred to a heated (30 °C) recording chamber. The chamber was perfused at a speed of 2–3 ml/min with artificial cerebrospinal fluid (ACSF) consisting of (in mM) 124 NaCl, 2.5 KCl, 2.5 CaCl_2_, 1.3 MgSO_4_, 26.2 NaHCO_3_, 1.25 NaH_2_PO_4_, and 11 glucose (saturated with 95% O_2_ and 5% CO_2_). To record NMDAR-mediated EPSCs (NMDAR-EPSCs), targeted neurons were voltage clamped at +40 mV. DNQX (20 μM) and picrotoxin (100 μM) were added to the ACSF. NMDAR-EPSCs were evoked by short current pulses (0.1 ms in duration) every 30 sec by extracellular stimulation of schaffer collaterals using a bipolar stimulating electrode. The stimulating electrode was placed at a distance of approximately 100 μm from the cell body. For determination of NMDA/AMPA receptor current ratios, the AMPA receptor current was quantified as the peak current recorded at Vh = −70 mV, and the NMDA receptor current was quantified 40 ms after stimulation onset at Vh = +40 mV. The recording pipette (3–5 MΩ) was filled with a solution containing (in mM) 102 Cs-gluconate, 3.7 NaCl, 11 BAPTA, 0.2 EGTA, 20 HEPES, 2 Mg-ATP, 0.3 Na_3_-GTP and 5 QX-314 (adjusted to pH 7.2 with CsOH). For t action potential (AP) and EPSP recordings, current-clamp recordings were made using an AxoPatch 200B amplifier (Molecular Devices, USA). The recording pipettes (3–5 MΩ) were filled with a solution containing (in mM): 145 K-gluconate, 5 NaCl, 1 MgCl_2_, 0.2 EGTA, 10 HEPES, 2 Mg-ATP, 0.1 Na_3_-GTP, and 10 phosphocreatine (adjusted to pH 7.2 with KOH). The spike activation protocol consisted of a sequence of 800-ms depolarization pulses from −40 pA to 200 pA, with a 20-pA step and a rate of 0.2 Hz. The membrane potential was continuously maintained at −65 mV by manual direct current injection. To assay E-S coupling, the initial slope of the EPSPs was measured for the first 2.5-ms period of the rising phase (in millivolts per millisecond). The neuron’s input resistance (Rin) was monitored continuously by applying a hyperpolarization current or voltage pulse throughout experiment, and neurons were discarded from analyses if Rin or Ra (access resistance) changed by 15% of their respective initial values or if the resting membrane potential changed by 5 mV or more. Data were collected using Clampex 9.2 (Molecular Devices, USA) at 5–10 kHz sample rate.

### Data statistics

Data were obtained or analyzed by the experimenter who was blind to treatments in the behavioral and electrophysiological experiments. Data were expressed as the means ± SEM, with p < 0.05 indicating significance. Statistical analysis of differences between two groups was tested by unpaired, two-tailed student’s t test (for example, the effect of GluN2B antagonist on freezing behavior in Fig. 1; the difference of the p-ERK and p-CREB level between two training paradigms in Fig. 4). For comparison of more than two groups, we used one-way ANOVA based on normality test if there was one independent variable (for example, changes in the amount and phosphorylation of membrane NMDAR subunits after fear conditioning in Fig. 2). We used two-way ANOVA if there were two independent variables (for example, the input-output analysis in Fig. 5; action potential number analysis in Fig. 6). We also used one-way ANOVA to statistically analyze the level of p-ERK and p-CREB at different time points after training (Fig. 4A,D). Student-Newman-Keuls test was used for multiple comparison post hoc tests using SigmaStat 3.2 (Systat Software, Inc.).

## Additional Information

**How to cite this article**: Sun, Y.-Y. *et al*. Surface expression of hippocampal NMDA GluN2B receptors regulated by fear conditioning determines its contribution to memory consolidation in adult rats. *Sci. Rep.* **6**, 30743; doi: 10.1038/srep30743 (2016).

## Supplementary Material

Supplementary Information
