# Supplementary material for: Surface expression of hippocampal NMDA GluN2B receptors regulated by fear conditioning determines its contribution to memory consolidation in adult rats
Source: Sci Rep. 2016 Aug 4;6:30743. doi: 10.1038/srep30743 (PMC4973269; doi:10.1038/srep30743)
Supplement: Supplementary Information [file srep30743-s1.pdf]

Surface expression of hippocampal NMDA GluN2B receptors regulated by fear conditioning determines its contribution to memory consolidation in adult rats

Yan-Yan Sun [1*], Wei Cai [1*], Jie Yu [1], Shu-Su Liu [1], Min Zhuo [3,4], Bao-Ming Li [1,2] and Xue-Han Zhang [1 CA]

[1]*Institute of Neurobiology and State Key Laboratory of Medical Neurobiology, Institutes of Brain Science, Fudan University, Shanghai 200032, China*

[2]*Center for Neuropsychiatric Diseases, Institute of Life Science, Nanchang University, Nanchang 330031, China*

[3] *Center for Neuron and Disease, Frontier Institutes of Science and Technology, Xi'an Jiaotong University, Xi'an 710049, China*

[4] *Department of Physiology, Faculty of Medicine, University of Toronto, 1 King's College Circle Toronto, Ontario M5S 1A8, Canada*

[*] *These two authors contributed equally.*

***Address correspondence to*:** Xue-Han Zhang, Ph.D.

Institute of Neurobiology,

Fudan University,

138 Yi Xue Yuan Road,

Shanghai 200032,

CHINA

Tel: 86-21-5423 7627

Fax: 86-21-5423 7647

Email: xuehanzhang@fudan.edu.cn

Supplementary Figure 1

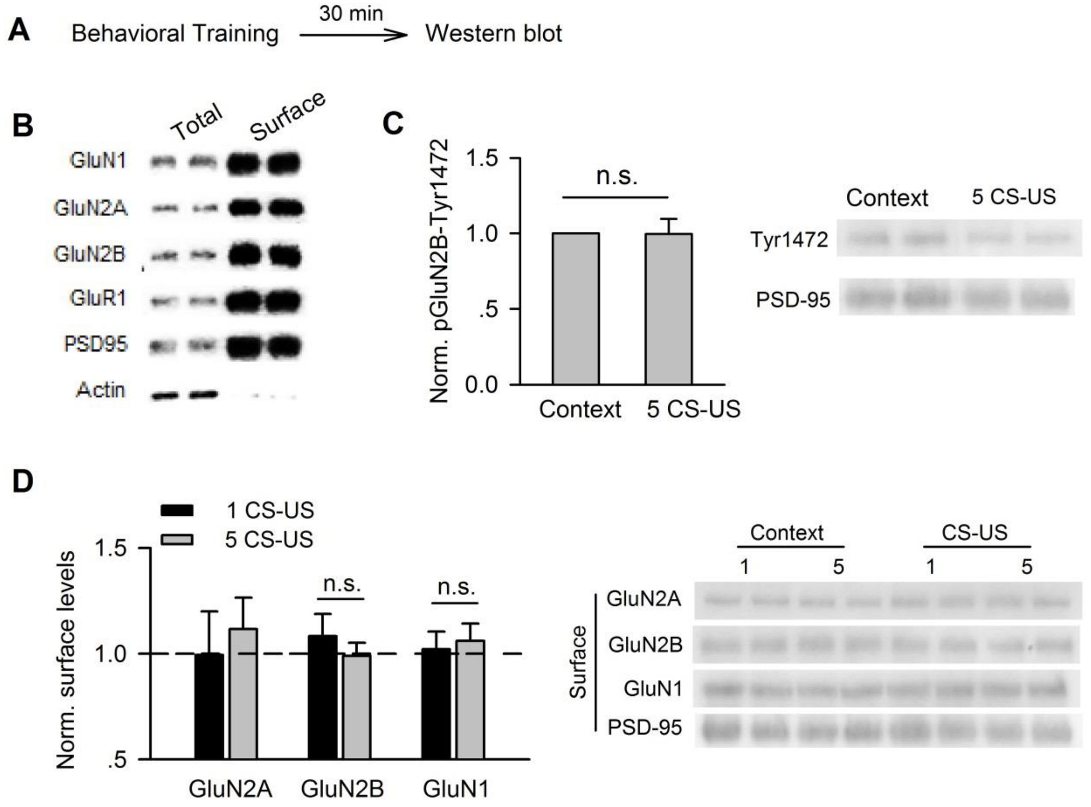

Figure 1. No changes in the phosphorylation at Tyr 1472 and amounts of membrane NMDAR examined at 30 min after fear conditioning.

(A) The graph depicts experiment design.

(B) Identification of membrane protein.

(C) and (D) Quantification analysis of surface pGluN2B at Tyr 1472 (*C*) and surface NMDAR subtypes (*D*) in membrane lysates of CA1 taken from context-control rats (context) versus rats conditioned with a single (1 CS-US) or five CS-US (5 CS-US) pairing examined at 30 min after conditioning, normalized to context control rats. n=3-5 rats for each group. *Right panels* indicate representative immunoblots.

Supplementary Figure 2

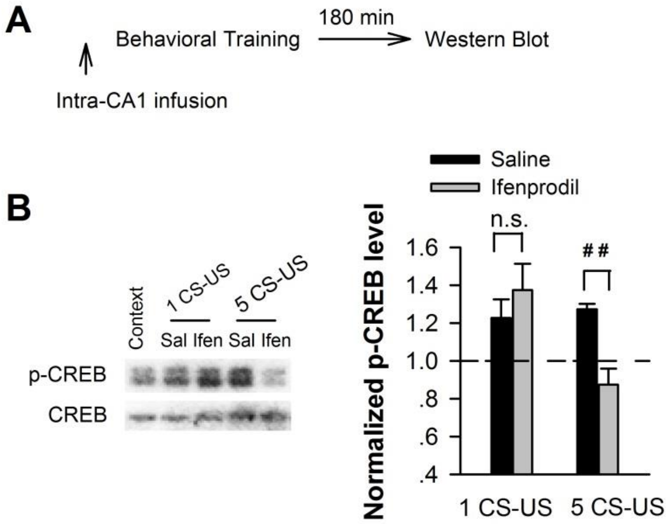

Figure 2. Effect of ifenprodil on CREB activity examined at 180 min after fear conditioning.

(A) The graph depicts experiment design.

(B) Immunoblots (*left*) and quantification analysis (*right)* of CREB phosphorylation (p-CREB) in CA1 lysates taken from context-control rats and conditioned rats with intra-CA1 infusion of ifenprodil versus saline prior to conditioning, normalized to context-control rats. $^{\#\#}p<0.01$. n=3 for each group.

Supplementary Figure 3

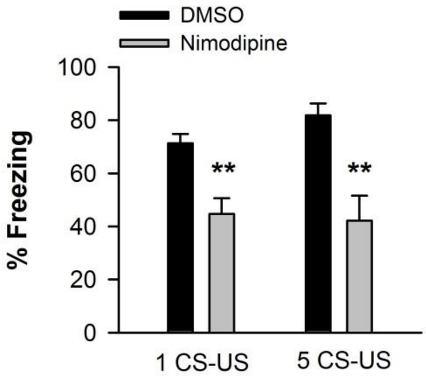

Figure 3. L-type Ca^{2+} channels are required for long-term memory elicited by both single and five CS-US conditioning in the CA1 region. Intra-CA1 infusion of L-type Ca^{2+} channel blocker nimodipine (0.34 μg) before fear conditioning and testing contextual fear memory at 48 h later. **$p<0.01$ versus DMSO, n=5-6 for each group.

Supplementary Figure 4

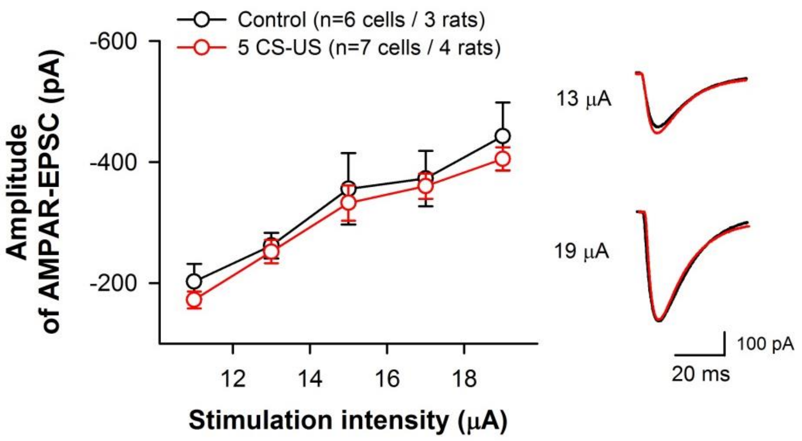

Figure 4. AMPAR functions did not change after fear conditioning. Summary for input-output curves of AMPAR-EPSCs in response to a series of stimulation intensities in CA1 pyramidal neurons from context-control rats versus conditioned rats (*left*). The CA1 slice was prepared at 10 min after fear conditioning. The AMPAR-EPSC traces represent the average of seven consecutive whole-cell recordings from a pyramidal neuron from a context-control rat (black trace) versus a five-CS-US-conditioned rat (red trace) (*right*).
